# On the Energy Efficiency of On-Off Keying Transmitters with Two Distinct Types of Batteries

**DOI:** 10.3390/s18041291

**Published:** 2018-04-23

**Authors:** Tingting Shen, Tao Wang, Yanzan Sun, Yating Wu, Yanliang Jin

**Affiliations:** Shanghai Institute for Advanced Communication and Data Science, Key laboratory of Specialty Fiber Optics and Optical Access Networks, Joint International Research Laboratory of Specialty Fiber Optics and Advanced Communication, Shanghai University, Shanghai 200444, China; caro121@shu.edu.cn (T.S.); ytwu@shu.edu.cn (Y.W.); wuhaide@shu.edu.cn (Y.J.)

**Keywords:** battery, energy efficiency, on-off keying, transmitters, sensors

## Abstract

As nodes in wireless sensor networks are usually powered by nonrenewable batteries, energy efficient design becomes critical. This paper considers a battery-powered transmitter using on-off keying (OOK) modulation and studies its energy efficiency in terms of the battery’s energy consumption for per bit transmission (BECPB). In particular, the transmitter may use one of two distinct types of batteries with battery utilization factor (BUF) depending on discharge current. The first has an instantaneous discharge current (IDC)-based BUF, while the second has a mean discharge current (MDC)-based BUF. For each type of battery, a closed-form BECPB expression is derived under a Rayleigh channel when a prescribed symbol error rate (SER) is guaranteed. Then theoretical analysis is made to study the impact of battery characteristic parameter γ, communication distance *d* and bandwidth *B* on the BECPB. Finally, the analysis is corroborated by numerical experimental results, which reveal that: the BECPB for each type of battery increases with γ and *d*; the BECPB for the two batteries first decreases and then increases with *B*, and there exists the optimal bandwidth corresponding to the minimum BECPB; the battery with IDC-based BUF corresponds to a larger BECPB. When γ and *d* are large, the BECPB for each type of battery is significantly higher than that for the ideal battery whose BUF is aways 1. For instance, when γ=0.015, d=90 m and B=10 kHz, the BECPB for IDC-based and MDC-based battery is nearly 60% amd 25% higher than that of the ideal battery, respectively.

## 1. Introduction

### 1.1. Background

With rapid development of microelectronic technology and wireless communications, Wireless Sensor Networks (WSNs) have drawn much attention in recent years [1]. WSNs are usually comprised of distributed sensor nodes to support many potential applications, including home networking, monitoring and health care, etc. In such scenarios, sensor nodes are typically powered by energy-limited batteries, which supply sensing, data processing and communication components [2]. In practice, the energy consumption of a sensor node is dominated by the communication components [3]. It has been shown that sensor node lifetime has a strong dependence on battery lifetime [1,2,3]. The malfunctioning of a few main nodes might lead to paralysis of the entire network. Hence, minimizing the sensor energy consumption is of great importance.

### 1.2. Related Work

Energy saving approaches have been proposed, including network protocols [1,2,4] as well as modulations schemes [3,5]. In [6], optimum transmission time and distance for best energy efficiency are analyzed for MQAM and MFSK, taking both transmission and circuit energy consumption into account. It is shown that the optimum total energy consumption per bit is higher for MFSK when *d* is small due to its circuit energy consumption. When *d* increases, MFSK becomes more energy-efficient than MQAM. In [7], the effect of channel bandwidth and active mode duration on energy consumption of modulation schemes including OOK, MPPM, MFSK, and MQAM are analyzed and compared over Rayleigh fading channel. Results show that OOK is more energy efficient for short range and ultra-wideband scenarios. Moreover, OOK is less complex in implementation compared with other schemes.

However, all the above works were carried out by assuming that the batteries are *ideal*, i.e., they have battery utilization factor (BUF) always equal to 1, where the BUF is defined as the ratio of the discharge current to the current actually consumed inside the battery. However, the BUF of a *realistic* battery is always smaller than 1, meaning that some energy is wasted during its discharge process. On this reason, the battery energy consumption depends not only on the energy-consuming modules, but also on the unique battery discharge characteristic described by the BUF [8,9,10,11].

Some research works have reported on the battery energy efficiency [12,13,14], which can be evaluated by the battery energy consumption for transmitting per message bit (BECPB). Clearly, increase of BECPB leads to degradation of battery energy efficiency. In [8], the BECPB is also derived base on the IDC-based BUF, considering the working mode of the circuit and integrating typical WSN transmission and reception modules with realistic battery models. In [9,10,11], the BECPB is studied for transmitters whose BUF depends on instantaneous discharge current (IDC), assuming the amplifier’s output current has the same waveform as the battery’s output current waveform.

Note that it is claimed in [15] that certain batteries may have a BUF dependent on the battery’s mean discharge current (MDC) over a period which can be as long as several seconds. However, the above characteristic has not been considered in previous works to the authors’ best knowledge.

### 1.3. Main Contributions

In this paper, we consider a battery-powered transmitter using on-off keying (OOK) modulation and study its battery energy efficiency in terms of BECPB. OOK is chosen because it features less complexity and cost than other modulation schemes such as MPPM, MFSK and MQAM. Moreover, OOK has an inherent mechanism to save energy, in the sense that the transmitter does not need to transmit signal when launching ‘0’. Due to the above reasons, OOK is suitable for portable devices and remains a strong candidate for realistic applications in WSNs. Moreover, the transmitter may use one of two distinct types of batteries. The first has an IDC-based BUF, while the second has a MDC-based BUF motivated by the claim made in [15]. Note that the above two types of BUF are applicable for Li-ion batteries as introduced in [15]. To be more specific, our contributions are summarized as follows:

- For each type of battery, the instantaneous battery power consumption is derived as a nonlinear function of the instantaneous battery discharge power and a battery characteristic parameter γ. It is also shown that this battery characteristic parameter depends on inherent properties of the battery. With the battery discharge power fixed, the instantaneous battery power consumption still increases as this battery characteristic parameter increases.
- For each type of battery, a closed-form BECPB expression is derived under Rayleigh channel when a prescribed symbol error rate (SER) is guaranteed. To the best of the authors’ knowledge, the BECPB for MDC-based BUF has not been reported in previous works.
- Theoretical analysis is made to show the impact of the battery characteristic parameter γ, communication distance and bandwidth on the BECPB. To the best of the authors’ knowledge, the above has not been studied in previous works.
- The analytical analysis is corroborated by numerical experimental results.

For readers’ convenience, symbols and their physical meanings frequently used later are summarized in Table 1. Acronyms used in the paper are summarized in Table 2.

## 2. Power of the Battery and Transmitter

**Figure 1:**
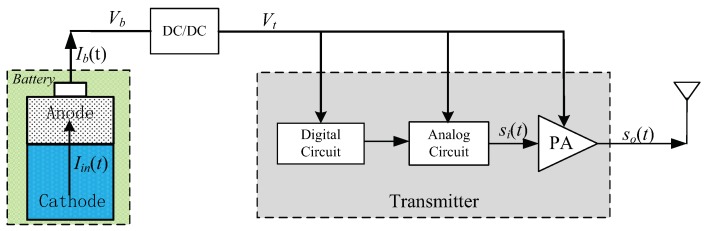
The battery-powered transmitter under study.

Figure 1 shows a diagram of a battery-powered transmitter under study. As shown there, the transmitter’s energy is supplied by a DC/DC convertor connected with a battery. Assume the convertor’s efficiency is η, we have

(1) $$\begin{array}{c} \eta =\frac{P_{\mathrm{tx}}\left(t\right)}{P_{\mathrm{out}}\left(t\right)} \end{array}$$

where $P_{\mathrm{tx}}\left(t\right)$ denotes the transmitter’s instantaneous power consumption, and $P_{\mathrm{out}}\left(t\right)$ is the battery’s instantaneous discharge power. We will establish power consumption models for $P_{\mathrm{tx}}\left(t\right)$, $P_{\mathrm{out}}\left(t\right)$ and $P_{\mathrm{bat}}\left(t\right)$ denoting the battery’s instantaneous power consumption in the following subsections.

### 2.1. Battery Power

We consider two types of batteries with different BUFs which can be described as follows.

*Battery with IDC-based BUF*: the battery’s instantaneous inner current is

(2) $$\begin{array}{c} I_{\mathrm{in}}\left(t\right)=\frac{I_{b}\left(t\right)}{\mu (I_{b}\left(t\right))} \end{array}$$

where $I_{b}\left(t\right)$ represents the instantaneous discharge current, $\mu \left(I_{b}\left(t\right)\right)=1-\omega I_{b}\left(t\right)$ is the BUF depending on $I_{b}\left(t\right)$, and ω is a BUF-related parameter. In such a case, the battery’s instantaneous power consumption is

(3) $$\begin{array}{c} P_{\mathrm{bat}}\left(t\right)=V_{b}I_{\mathrm{in}}\left(t\right)=\frac{P_{\mathrm{out}}\left(t\right)}{1-\gamma P_{\mathrm{out}}\left(t\right)} \end{array}$$

where $V_{b}$ is the battery’s discharge voltage, $P_{\mathrm{out}}\left(t\right)=V_{b}I_{b}\left(t\right)$ and

(4) $$\begin{array}{c} \gamma =\omega /V_{b} \end{array}$$

is the battery characteristic parameter connecting $P_{\mathrm{bat}}\left(t\right)$ with $P_{\mathrm{out}}\left(t\right)$. From the above equations, it can be seen that even when $P_{\mathrm{out}}\left(t\right)$ is fixed, $P_{\mathrm{bat}}\left(t\right)$ still increases as γ increases.

*Battery with MDC-based BUF*: the battery’s instantaneous inner current is

(5) $$\begin{array}{c} I_{\mathrm{in}}\left(t\right)=\frac{I_{b}\left(t\right)}{\mu (I_{\mathrm{mean}})} \end{array}$$

where $I_{\mathrm{mean}}$ denotes the average of $I_{b}\left(t\right)$ over the discharge process, $\mu \left(I_{\mathrm{mean}}\right)=1-\omega I_{\mathrm{mean}}$ is the $I_{\mathrm{mean}}$-dependent BUF. In such a case, the battery’s instantaneous power consumption is

(6) $$\begin{array}{c} P_{\mathrm{bat}}\left(t\right)=V_{b}I_{\mathrm{in}}\left(t\right)=\frac{P_{\mathrm{out}}\left(t\right)}{1-\gamma P_{\mathrm{mean}}} \end{array}$$

where $P_{\mathrm{mean}}$ represents the average of $P_{\mathrm{out}}\left(t\right)$ over the discharge process. From the above equations, it can be seen that even when $P_{\mathrm{out}}\left(t\right)$ and $P_{\mathrm{mean}}$ are fixed, $P_{\mathrm{bat}}\left(t\right)$ still increases as γ increases.

Note that for the above two types of batteries, γ is strictly greater than 0, thus $P_{\mathrm{bat}}\left(t\right)$ is strictly greater than $P_{\mathrm{out}}\left(t\right)$, meaning that the above two batteries always has an energy waste during discharge process. When γ=0, $I_{\mathrm{in}}\left(t\right)$ is always equal to $I_{b}\left(t\right)$ and $P_{\mathrm{bat}}\left(t\right)$ is always equal to $P_{\mathrm{out}}\left(t\right)$, which corresponds to an ideal battery without energy waste during discharge process.

### 2.2. Transmitter Power

Clearly, $P_{\mathrm{tx}}\left(t\right)$ is the sum power consumption of all transmitter components, which consists of the following parts:

- a digital circuit responsible for digital signal processing that transforms sampled information to digital bits, and controlling other circuits in the transmitter. The digital circuit remains active and consumes a fixed power denoted by $P_{d}$ independently of emitted symbols.
- an analog circuit which usually comprises baseband or radio frequency analog circuits such as a voltage controlled oscillator and filters. Specifically, the analog circuit is controlled by the digital circuit to generate a sinusoidal waveform and delivers it to the power amplifier when emitting a symbol ‘1’, but to be switched off when emitting a symbol ‘0’. When emitting ‘1’, we assume the analog circuit consumes a fixed power denoted by $P_{a}$.
- a power amplifier (PA) which feeds the antenna with OOK-modulated signal expressed as
  (7) $$\begin{array}{c} s_{o}\left(t\right)=\left\{\begin{array}{cc} \sqrt{2P_{1}}sin\left(2\pi f_{c}t\right) & \;\;\mathrm{when}\;\;\;\mathrm{emitting}\;\;\mathrm{symbol}\;\;\;‘1’,\\ 0 & \;\;\mathrm{when}\;\;\;\mathrm{emitting}\;\;\mathrm{symbol}\;\;\;‘0’, \end{array}\right. \end{array}$$
  where $P_{1}$ denotes the average signal power for emitting symbol ‘1’.

**Figure 2:**
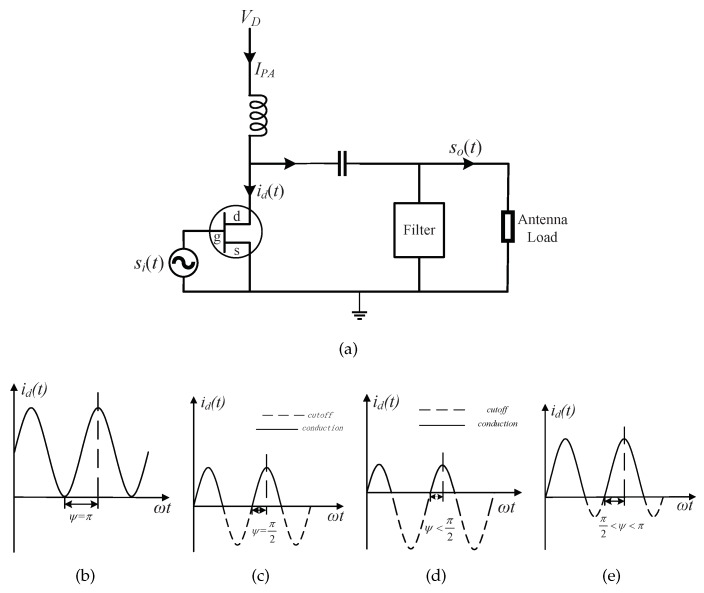
Illustrations for the low-cost power amplifier. (**a**) a diagram of the power amplifier; (**b**) class A; (**c**) class B; (**d**) class C; (**e**) class AB.

A diagram of commercially available low-cost PA is shown in Figure 2a, where a transistor is used. Its operation principle and power consumption is explained in details in the following.

When symbol ‘0’ is emitted, the PA can be turned off by the digital circuit. When symbol ‘1’ is emitted, a sinusoidal waveform is generated by the analog circuit and input to the PA, and this waveform can be added with a quiescent bias, so as to make the transistor conducted within a whole or part of each sinusoidal waveform’s cycle. As elaborated by pages 39–41 in [16], the degree of conduction is characterized by the so-called conduction angle ψ, and the PA can be classified into class A (ψ=π), B($\psi =\frac{\pi }{2}$), C($\psi <\frac{\pi }{2}$), and AB($\frac{\pi }{2}<\psi <\pi$) according to the specific value for ψ as illustrated in Figure 2b,c.

To establish a power consumption model for the PA, note that the current $I_{\mathrm{PA}}$ drawn from the power supply is stable and equal to the DC component of the total current $i_{d}\left(t\right)$ flowing into the transistor, while the current $s_{o}\left(t\right)$ driving the antenna load is equal to the first-order harmonic component of $i_{d}\left(t\right)$, while other higher-order harmonic components are eliminated by the filter. According to the derivations made on pages 39–41 of [16], the power loss factor, defined as the ratio of the PA’s power dissipation to the radiated signal power, is expressed as

(8) $$\begin{array}{c} \alpha =\frac{1}{\eta_{\mathrm{PA}}\left(\psi \right)}-1 \end{array}$$

where

(9) $$\begin{array}{c} \eta_{\mathrm{PA}}\left(\psi \right)=\frac{2\psi -sin(2\psi )}{4(sin(\psi )-\psi cos(\psi ))}. \end{array}$$

In summary, the PA’s power consumption is

(10) $$\begin{array}{c} P_{\mathrm{PA}}\left(t\right)=\left\{\begin{array}{cc} \left(1+\alpha \right)P_{1} & \;\;\mathrm{when}\;\;\mathrm{emitting}\;\;\mathrm{symbol}\;\;‘1’\\ 0 & \;\;\mathrm{when}\;\;\mathrm{emitting}\;\;\mathrm{symbol}\;\;‘0’ \end{array}\right. \end{array}$$

Summarizing the above power models for all components, the discharge power from the battery is

(11) $$\begin{array}{c} P_{\mathrm{out}}\left(t\right)=\frac{P_{\mathrm{tx}}\left(t\right)}{\eta }=\left\{\begin{array}{cc} \frac{\left(1+\alpha \right)P_{1}+P_{a}+P_{d}}{\eta } & \;\;\mathrm{when}\;\;\mathrm{emitting}\;\;‘1’\\ \frac{P_{d}}{\eta } & \;\;\mathrm{when}\;\;\mathrm{emitting}\;\;‘0’ \end{array}\right. \end{array}$$

## 3. Derivation of the BECPB for Rayleigh Channel

This section will derive BECPB for the OOK transmitter to guarantee a prescribed SER when a noncoherent detector is used at a receiver, and a Rayleigh fading channel is assumed between the transmitter and the receiver.

### 3.1. Battery Discharge Power for Prescribed SER

Assume the channel gain is given by $\frac{1}{L(d)}$, where L(d) represents the path loss expressed as

(12) $$\begin{array}{c} L\left(d\right)=M_{l}G_{0}d^{k}, \end{array}$$

where *d* is the transmitter-receiver distance, *k* is the power-decay exponent, $G_{0}$ and $M_{l}$ denote the path loss at 1 m and the link margin, respectively.

For Rayleigh fading channel, it can be shown that the average SER is expressed as

(13) $$\begin{array}{c} SER=\frac{1}{\rho +2} \end{array}$$

where ρ represents the average received SNR written as (refer to Equation (30) in [7]).

(14) $$\begin{array}{c} \rho =\frac{P_{1}/L\left(d\right)}{2N_{0}B}. \end{array}$$

Assume the SER must be smaller than a prescribed value Γ. It can be shown that

(15) $$\begin{array}{c} P_{1}=2N_{0}M_{l}G_{0}\left(\frac{1}{\Gamma }-2\right)Bd^{k}, \end{array}$$

and after plugging it into Equation (11),the discharge power from the battery is

(16) $$\begin{array}{c} P_{\mathrm{out}}\left(t\right)=\left\{\begin{array}{cc} \frac{f_{\mathrm{ray}}\left(B,d\right)+P_{a}+P_{d}}{\eta } & \;\mathrm{when}\;\;\mathrm{emitting}\;\;\mathrm{symbol}\;\;‘1’\\ \frac{P_{d}}{\eta } & \;\mathrm{when}\;\;\mathrm{emitting}\;\;\mathrm{symbol}\;\;‘0’, \end{array}\right. \end{array}$$

where

(17) $$\begin{array}{c} f_{\mathrm{ray}}\left(B,d\right)=\xi Bd^{k} \end{array}$$

with $\xi =2\left(1+\alpha \right)N_{0}M_{l}G_{0}\left(\frac{1}{\Gamma }-2\right)$.

### 3.2. BECPB for Battery with IDC-Based BUF

After plugging Equation (16) into Equation (3), the power consumption for battery with IDC-based BUF is

(18) $$\begin{array}{c} P_{\mathrm{bat}}\left(t\right)=\left\{\begin{array}{cc} \frac{f_{\mathrm{ray}}\left(B,d\right)+P_{a}+P_{d}}{\eta -\gamma [f_{\mathrm{ray}}\left(B,d\right)+P_{a}+P_{d}]} & \;\;\mathrm{when}\;\;\mathrm{emitting}\;\;\mathrm{symbol}\;\;‘1’\\ \frac{P_{d}}{\eta -\gamma P_{d}} & \;\;\mathrm{when}\;\;\mathrm{emitting}\;\;\mathrm{symbol}\;\;‘0’, \end{array}\right. \end{array}$$

As a symbol duration is $\frac{1}{B}$ and symbol ‘1’ and ‘0’ are transmitted with the same probability, the BECPB for the battery with IDC-based BUF in Rayleigh channel is

(19) $$\begin{array}{c} E_{\mathrm{bat},I}=\frac{1}{2B}\left[\frac{f_{\mathrm{ray}}\left(B,d\right)+P_{a}+P_{d}}{\eta -\gamma [f_{\mathrm{ray}}\left(B,d\right)+P_{a}+P_{d}]}+\frac{P_{d}}{\eta -\gamma P_{d}}\right] \end{array}$$

### 3.3. BECPB for Battery with MDC-Based BUF

Substituting Equation (16) into Equation (6), the power consumption for the battery with MDC-based BUF is given by

(20) $$\begin{array}{c} P_{\mathrm{bat}}\left(t\right)=\left\{\begin{array}{cc} \frac{f_{\mathrm{ray}}\left(B,d\right)+P_{a}+P_{d}}{\eta (1-\gamma P_{\mathrm{mean}})} & \;\;\mathrm{when}\;\;\mathrm{emitting}\;\;\mathrm{symbol}\;\;‘1’\\ \frac{P_{d}}{\eta (1-\gamma P_{\mathrm{mean}})} & \;\;\mathrm{when}\;\;\mathrm{emitting}\;\;\mathrm{symbol}\;\;‘0’ \end{array}\right. \end{array}$$

where $P_{\mathrm{mean}}$ is the average of $P_{\mathrm{out}}\left(t\right)$ and can be expressed as

(21) $$\begin{array}{c} P_{\mathrm{mean}}=\frac{f_{\mathrm{ray}}\left(B,d\right)+P_{a}+2P_{d}}{2\eta } \end{array}$$

In the same way used in the former section, the BECPB for the battery with MDC-based BUF in Rayleigh channel is

(22) $$\begin{array}{cc} E_{\mathrm{bat},M} & =\frac{1}{2B}\frac{f_{\mathrm{ray}}\left(B,d\right)+P_{a}+2P_{d}}{\eta -\gamma \frac{f_{\mathrm{ray}}\left(B,d\right)+P_{a}+2P_{d}}{2}} \end{array}$$

Note that an ideal battery has a BUF always equal to 1. Moreover, it can be easily seen that the BECPB for the ideal battery can be evaluated by either Equation (19) or Equation (22) by setting γ as 0. As a result, it can be readily shown that the BECPB for the ideal battery is

(23) $$\begin{array}{c} E_{\mathrm{bat},\mathrm{ideal}}=\frac{1}{2B}\frac{\xi Bd^{k}+P_{a}+2P_{d}}{\eta }. \end{array}$$

Since both Equations (19) and (22) are increasing with γ, γ=0 corresponds to the smallest BECPB, meaning that the battery with either IDC or MDC base BUF corresponds to a larger BECPB than the ideal battery, and the gap increases as γ increases.

## 4. Theoretical Analysis

This section will analyze the impact of battery characteristic parameter, distance and bandwidth on the BECPB for different types of batteries, and compare the BECPB for different types of batteries theoretically.

### 4.1. Impact of γ on BECPB

From Equation (19), it can be shown that its first part increases with γ, so is the second part, which means that $E_{\mathrm{bat},I}$ is an increasing function of γ. Similar to Equation (19), $E_{\mathrm{bat},M}$ increases with γ as well.

To sum up, the BECPBs for both types of batteries are increasing function of γ. This is due to the fact that higher γ leads to smaller BUF and thus more energy waste during battery discharge process.

### 4.2. Impact of *d* on BECPB

According to Equation (19), it can be shown that its first part increases with *d*, while the second part is a constant. Hence, $E_{\mathrm{bat},I}$ is an increasing function of *d*. Similarly, $E_{\mathrm{bat},M}$ also increases as *d* increases.

To sum up, the BECPBs for both types of batteries increase with *d*. The reason behind this phenomenon is that the transmission energy depends on the transmission distance *d* and it increases with *d*.

Specifically, for the ideal battery, it can be shown by Equation (23) that its BECPB increase with γ.

### 4.3. Impact of *B* on BECPB

From Equation (19), the derivative of $E_{\mathrm{bat},I}$ with respect to *B* is given by

(24) $$\begin{array}{c} E_{\mathrm{bat},I}^{\prime }=\frac{\gamma \lambda^{2}B^{2}+2v\lambda \gamma B-v\left(\eta -\gamma v\right)}{2B^{2}{\left[\eta -\gamma \left(\lambda B+v\right)\right]}^{2}}-\frac{P_{d}}{2B^{2}\left(\eta -\gamma P_{d}\right)} \end{array}$$

where $\lambda =\xi d^{k}$, $v=P_{a}+P_{d}$. Note that $\frac{P_{d}}{2B^{2}\left(\eta -\gamma P_{d}\right)}$ is much smaller than the first term in Equation (24), therefore we can make the approximation:

(25) $$\begin{array}{c} E_{\mathrm{bat},I}^{\prime }\approx \frac{\gamma \lambda^{2}B^{2}+2v\lambda \gamma B-v\left(\eta -\gamma v\right)}{2B^{2}{\left[\eta -\gamma \left(\lambda B+v\right)\right]}^{2}}. \end{array}$$

After mathematical derivation, it can be shown that the equation $E_{\mathrm{bat},I}^{\prime }=0$ has two solutions expressed as

(26) $$\begin{array}{c} B_{1,I}=\frac{-\gamma v-\sqrt{\gamma v\eta }}{\gamma \lambda }<0,B_{2,I}=\frac{-\gamma v+\sqrt{\gamma v\eta }}{\gamma \lambda }>0. \end{array}$$

When $0<B<B_{2,I}$, $E_{\mathrm{bat},I}^{\prime }<0$, $E_{\mathrm{bat},I}$ decreases with *B*. When $B>B_{2,I}$, $E_{\mathrm{bat},I}^{\prime }>0$, $E_{\mathrm{bat},I}$ increases with *B*. As a result, $B_{2,I}$ is the optimal bandwidth leading to the smallest BECPB for the battery with IDC-based BUF.

From Equation (22), the derivative of $E_{\mathrm{bat},M}$ with respect to *B* is

(27) $$\begin{array}{c} E_{\mathrm{bat},M}^{\prime }=\frac{\gamma \lambda^{2}B^{2}+2w\lambda \gamma B-w\left(2\eta -\gamma w\right)}{4B^{2}{\left[\eta -\frac{\gamma }{2}\left(\lambda B+w\right)\right]}^{2}}, \end{array}$$

where $\lambda =\xi d^{k}$,$w=P_{a}+2P_{d}$. It can be derived that the equation $E_{\mathrm{bat},M}^{\prime }=0$ has two solutions written as

(28) $$\begin{array}{c} B_{1,M}=\frac{-\gamma w-\sqrt{2\gamma w\eta }}{\gamma \lambda }<0,B_{2,M}=\frac{-\gamma w+\sqrt{2\gamma w\eta }}{\gamma \lambda }>0. \end{array}$$

When $0<B<B_{2,M}$, $E_{\mathrm{bat},M}^{\prime }<0$, $E_{\mathrm{bat},M}$ decreases with *B*. When $B>B_{2,M}$, $E_{\mathrm{bat},M}^{\prime }>0$, $E_{\mathrm{bat},M}$ increases with *B*. As a result, $B_{2,M}$ is the optimal bandwidth corresponding to the smallest BECPB for the battery with MDC-based BUF.

We will compare the optimal bandwidth of the two types of batteries. The difference between them can be expressed as

(29) $$\begin{array}{cc} \Delta B=B_{2,M}-B_{2,I} & =\frac{-\gamma w+\sqrt{2\gamma w\eta }}{\gamma \lambda }-\frac{-\gamma v+\sqrt{\gamma v\eta }}{\gamma \lambda }\\ & =\frac{-\gamma \left(w-v\right)+\sqrt{\gamma \eta }\left(\sqrt{2w}-\sqrt{v}\right)}{\gamma \lambda }\\ & \geq \frac{-\gamma \left(w-v\right)+\sqrt{\gamma \eta }\left(\sqrt{w}-\sqrt{v}\right)}{\gamma \lambda }\\ & =\frac{\sqrt{\gamma }\left(\sqrt{w}-\sqrt{v}\right)\left[\sqrt{\eta }-\sqrt{\gamma }\left(\sqrt{w}+\sqrt{v}\right)\right]}{\gamma \lambda }. \end{array}$$

where $B_{2,M}$ and $B_{2,I}$ are the optimal bandwidth for battery with MDC-based BUF and IDC-based BUF, respectively. Note that w>v and $\sqrt{w}+\sqrt{v}<<1$, thus $\sqrt{\eta }-\sqrt{\gamma }\left(\sqrt{w}+\sqrt{v}\right)>0$, ΔB>0. That is to say, the optimal bandwidth for the battery with MDC-based BUF is always larger.

As can be shown by Equation (23), the BECPB for the ideal battery decreases with *B*.

### 4.4. Comparison of the BECPB for Different Batteries

To facilitate the BECPB comparison between the two batteries, the difference between the BECPBs is given by

(30) $$\begin{array}{cc} \Delta E_{\mathrm{bat}}=E_{\mathrm{bat},I}-E_{\mathrm{bat},M} & =\frac{1}{2B}\left[\frac{\frac{1}{2}\gamma t_{1}t_{2}-\eta P_{d}}{\left(\eta -\gamma t_{1}\right)\left(\eta -\frac{1}{2}\gamma t_{2}\right)}+\frac{P_{d}}{\eta -\gamma P_{d}}\right] \end{array}$$

(31) $$\begin{array}{cc} & \;=\frac{1}{2B}\frac{-\gamma \eta P_{d}\left(t_{1}+\frac{1}{2}t_{2}\right)+\frac{1}{2}\gamma \eta t_{1}t_{2}+\gamma \eta P_{d}^{2}}{\left(\eta -\gamma P_{d}\right)\left(\eta -\gamma t_{1}\right)\left(\eta -\frac{\gamma }{2}t_{2}\right)}. \end{array}$$

where $t_{1}=\xi Bd^{k}+P_{a}+P_{d}$, $t_{2}=\xi Bd^{k}+P_{a}+2P_{d}$. Note that $P_{d}<<1$, thus $t_{1}\approx t_{2}$ and $-\gamma \eta P_{d}\left(t_{1}+\frac{1}{2}t_{2}\right)+\frac{1}{2}\gamma \eta t_{1}t_{2}+\gamma \eta P_{d}^{2}\approx -\frac{3}{2}\gamma \eta P_{d}t_{1}+\frac{1}{2}\gamma \eta t_{1}^{2}$, as a result, $\Delta E_{\mathrm{bat}}=0$ has two solutions, namely $d_{1}={\left(\frac{2P_{d}-P_{a}}{\xi B}\right)}^{-k}\approx 0$ and $d_{2}=0$. Therefore when d>0, $\Delta E_{\mathrm{bat}}>0$, which means the battery with IDC-based BUF corresponds to a higher BECPB than the battery with MDC-based BUF.

## 5. Simulation Results

This section shows numerical results using realistic parameters. We will first describe system setup, and then show results to corroborate the above theoretical analysis.

### 5.1. System Setup

The system parameters used in simulation experiments are listed in Table 3. A Class-A PA is used at the transmitter (ψ=π), thus $\eta_{PA}=0.5$ and α=1. In practice, the bandwidth *B* is set to be no more than 30 kHz, and the SER is set as $\Gamma =10^{-3}$ for Rayleigh fading channel.

### 5.2. Impact of γ on BECPB

**Figure 3:**
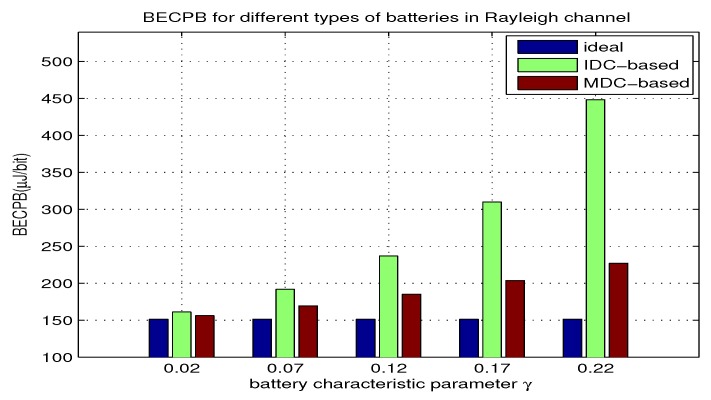
BECPB for two types of batteries over γ.

In this part, we use equation Equations (19) and (22) to compute BECPB when γ increases from 0.02 to 0.22 with d=50 m and B=10 kHz for the two types of batteries. The results are shown in Figure 3. From the results, we can observe the impact of γ on the BECPB, and compare the BECPB for different batteries. Moreover, we can justify if the theoretical analysis agrees with the evaluated results.

Specifically, the following points can be observed:

- When γ is fixed, the battery with IDC-based BUF corresponds to a higher BECPB than that with MDC-based BUF. As γ increases, both BECPBs and their gap increase. As shown in Figure 3, the BECPB for the battery with IDC-based BUF is about 18% higher than that with MDC-based BUF when γ=0.07.
- Each realistic battery corresponds to a higher BECPB than the ideal one, and with the increase of γ, the BECPB gap between the two batteries and the ideal one gradually expands. In particular, when γ=0.07, the BECPB for battery with IDC-based BUF is about 35% higher than that for the ideal one, meanwhile the BECPB for battery with MDC-based BUF is about 15% higher. The above observations corroborate the theoretical analysis made in Section 4.1.

### 5.3. Impact of *d* on BECPB

**Figure 4:**
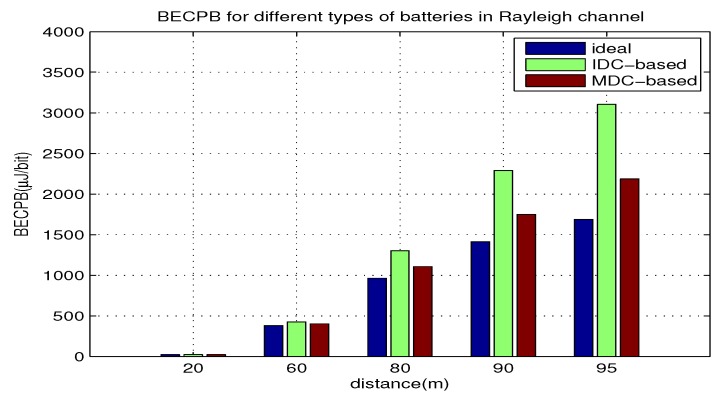
BECPB for two types of batteries over *d*.

In this part, we use equation Equations (19) and (22) to compute BECPB when *d* increases from 20 m to 95 m with γ=0.015 and B=10 kHz for the two types of batteries. The results are shown in Figure 4. From the results, we can see the impact of *d* on the BECPB, and compare the BECPB for different batteries. Moreover, we can justify if the theoretical analysis agrees with the evaluated results. The following points can be observed:

- When *d* is fixed, the BECPB for the battery with IDC-based BUF is higher than that with MDC-based BUF. As *d* increases, BECPBs for both types and their gap increase. When d=90 m, the BECPB for the battery with IDC-based BUF is about 30% higher than that with the MDC-based BUF.
- Clearly, the BECPB for the two batteries is larger than that for the ideal battery. With the increase of *d*, the BECPB gap between each battery and the ideal one gradually expands. When d=90 m, the BECPB for the battery with IDC-based BUF and MDC-based BUF is about 60% and 25% higher than the ideal battery, respectively. The above observations support the theoretical analysis made in Section 4.2.

### 5.4. Impact of *B* on BECPB

**Figure 5:**
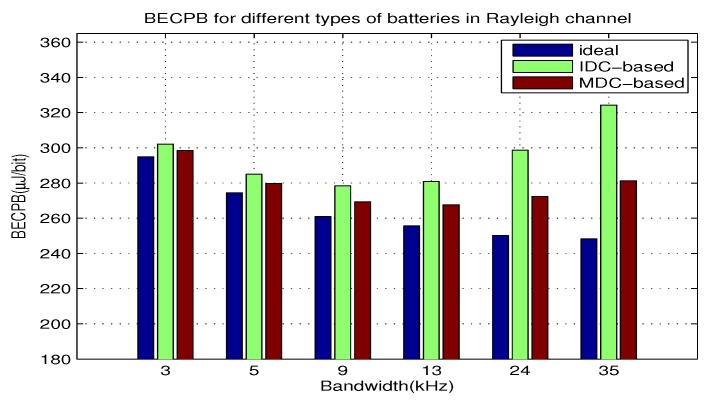
BECPB for two types of batteries over *B*.

In this part, we use equation Equation (19) and Equation (22) to compute BECPB when *B* increases from 3 kHz to 35 kHz with γ=0.015 and d=60 m for the two types of batteries. The results are shown in Figure 5. From the results, we can see the impact of *B* on the BECPB, and compare the BECPB for different batteries. Moreover, we can justify if the theoretical analysis agrees with the evaluated results.

The following points can be observed:

- The BECPB for the ideal battery decreases with *B*, while the BECPB for each realistic battery first decreases and then increases with *B*. There exists the optimal bandwidth corresponding to the minimum BECPB.
- The BECPB for each realistic batteries is always larger than that for the ideal battery. With the increase of *d*, the BECPB gap gradually expands.
- The optimal bandwidth for the battery with IDC-based BUF is 9kHz, which is smaller than that with MDC-based BUF, namely 13kHz. When B=9 kHz, the BECPB for the battery with IDC-based BUF is 8% higher than the ideal battery. Similarly, the BECPB for the battery with MDC-based BUF is about 6% higher when B=13 kHz. The above observations are consistent with the theoretical analysis made in Section 4.3.

## 6. Conclusions

In this paper, the BECPBs of OOK transmitters using realistic batteries are derived and compared. Two types of batteries with different BUFs are considered, with one depending on the instantaneous discharge current, while the other depending on the mean discharge current. Results show that the BECPB is related not only to the transmission and circuit energy consumption, but also to the battery characteristic parameter γ. Specifically, the BECPB for each type of battery increases with γ and distance; the BECPBs for the two nonlinear batteries first decrease and then increase with bandwidth. There exists the optimal bandwidth corresponding to the minimum BECPB; the battery with IDC-based BUF corresponds to a higher BECPB than the one with MDC-based BUF. When γ and distance are large, the nonlinear effect of battery has significant influence on BECPB, which cannot be ignored. The comparative study of the energy consumption of the two battery utilization models can provide guidance in energy-efficient design of wireless sensor nodes.

## Figures and Tables

**Table 1:** Symbol definitions and physical meanings.

Symbol Definitions	Physical Meaning
$V_{b}$	battery output voltage
$I_{b}$	discharge current of battery
ω	BUF-related parameter
$P_{\mathrm{out}}\left(t\right)$	instantaneous discharge power of battery
$P_{\mathrm{mean}}$	mean discharge power of battery
$P_{\mathrm{bat}}\left(t\right)$	instantaneous power consumption of battery
$\gamma =\frac{\omega }{V_{b}}$	battery characteristic parameter connecting $P_{\mathrm{bat}}\left(t\right)$ with $P_{\mathrm{out}}\left(t\right)$
$P_{d}$	digital circuit power consumption
$P_{a}$	RF circuit power consumption
*d*	transmitter-receiver distance
*η*	DC/DC convertor efficiency
*k*	path-loss decay exponent
$N_{0}$	single-sided power spectral density of AWGN
$M_{l}$	link margin
$G_{0}$	channel path loss at 1 meter
$E_{\mathrm{bat},I}$	BECPB for IDC-based BUF
$E_{\mathrm{bat},M}$	BECPB for MDC-based BUF

**Table 2:** Summary of acronyms used in the paper.

Acronyms	Full Name
WSNs	Wireless Sensor Networks
OOK	On-Off Keying
BECPB	battery energy consumption per message bit
BUF	battery utilization factor
IDC	instantaneous discharge current
MDC	mean discharge current
SER	symbol error rate

**Table 3:** Parameters used in the numerical experiments.

*k*	$M_{l}$	$G_{0}$	$P_{a}$	$P_{d}$	η	α	$N_{0}$	$V_{b}$	Γ
3.2	40 dB	27 dB	225 mW	10 mW	0.8	1	−164 dBm	3.7 V	$10^{-3}$
